# Clinical Performance and Safety of 108 SpineJack Implantations: 1-Year Results of a Prospective Multicentre Single-Arm Registry Study

**DOI:** 10.1155/2015/173872

**Published:** 2015-12-30

**Authors:** David Noriega, Gianluca Maestretti, Christian Renaud, Natale Francaviglia, Mourad Ould-Slimane, Steffen Queinnec, Helmut Ekkerlein, Frank Hassel, Rainer Gumpert, Pascal Sabatier, Hervé Huet, Miguel Plasencia, Nicolas Theumann, Alexander Kunsky, Antonio Krüger

**Affiliations:** ^1^Valladolid University Hospital, Royal Academy of Medicine and Surgery, 47008 Valladolid, Spain; ^2^Department of Orthopedic Surgery, Spine Unit, HFR Fribourg-Cantonal Hospital, 1708 Fribourg, Switzerland; ^3^Department of Orthopedics, Toulouse Lautrec Clinic, 81030 Albi, France; ^4^Department of Neurosurgery, A.R.N.A.S. Civico Di Cristina Benfratelli, 90127 Palermo, Italy; ^5^Department of Orthopedic Surgery, University Hospital of Rouen, 76031 Rouen, France; ^6^Department of Orthopedic Surgery, Hospital Beaujon, 92110 Clichy, France; ^7^Department of Trauma and Orthopedic Surgery, Südostbayern AG Clinic, Traunstein Clinic, 83278 Traunstein, Germany; ^8^Department of Spine Surgery, Loretto Hospital, 79100 Freiburg, Germany; ^9^LKH-Univ. Klinikum Graz, 8036 Graz, Austria; ^10^Department of Neurosurgery, Clinic of Cèdres, 31700 Cornebarrieu, France; ^11^Department of Neuroradiology, Regional University Hospital, 14033 Caen, France; ^12^Department of Traumatology and Orthopedic Surgery, University Hospital Principe De Asturias, 28805 Madrid, Spain; ^13^Department of Radiology, Bois-Cerf Clinic, 1006 Lausanne, Switzerland; ^14^Department of Neurosurgery Spine, Private Hospital Sévigné, 35567 Cesson-Sévigné, France; ^15^Department of Trauma and Reconstructive Surgery, Philipps University of Marburg, University Hospital of Giessen and Marburg, 35043 Marburg, Germany

## Abstract

This prospective, consecutive, multicentre observational registry aimed to confirm the safety and clinical performance of the SpineJack system for the treatment of vertebral compression fractures (VCF) of traumatic origin. We enrolled 103 patients (median age: 61.6 years) with 108 VCF due to trauma, or traumatic VCF with associated osteoporosis. Primary outcome was back pain intensity (VAS). Secondary outcomes were Oswestry Disability Index (ODI), EuroQol-VAS, and analgesic consumption. 48 hours after surgery, a median relative decrease in pain intensity of 81.5% was observed associated with a significant reduction in analgesic intake. Improvements in disability (91.3% decrease in ODI score) and in quality of life (increase 21.1% of EQ-VAS score) were obtained 3 months after surgery. All results were maintained at 12 months. A reduction in the kyphotic angulation was observed postoperatively (−5.4 ± 6.3°; *p* < 0.001), remained at 12 months (−4.4 ± 6.0°, *p* = 0.002). No adverse events were implant-related and none required device removal. Three patients (2.9%) experienced procedure-related complications. The overall adjacent fracture rate up to 1 year after surgery was 2.9%. The SpineJack procedure is an effective, low-risk procedure for patients with traumatic VCF allowing a fast and sustained improvement in quality of life over 1 year after surgery.

## 1. Introduction

Vertebroplasty and kyphoplasty are minimally invasive accepted procedures in the therapy of painful osteoporotic compression fractures [1–5]. Both techniques are indicated after inefficient conservative pain therapy in “stable” vertebral compression fractures (VCF). Acute traumatic fractures have to be differentiated. Traumatic fractures are related to acute trauma and can be witnessed also in osteoporotic patients. The involvement of the vertebra's posterior wall is a relative contraindication. Theoretical risks of posterior wall displacement are potential cement leakages into the spinal canal and the further dislocation of bone fragments into the spinal canal. Both incidents could lead to neurological deficits [3, 5].

Balloon kyphoplasty has been used for the treatment of fresh traumatic fractures [6, 7]. Several clinical and biomechanical studies have shown that there is a height loss after deploying the balloons [8, 9]. To improve anatomical restoration of the endplates of the vertebral body (VB) a new intravertebral reduction device was designed. The aim of this study was to confirm the safety and clinical performance of the SpineJack system in combination with Cohesion bone cement for the treatment of traumatic VCF. The study protocol calls for a final analysis of the results after a 2-year postoperative follow-up for all patients. We present 1-year results focusing on 103 patients assessed peroperatively and 48 h postoperatively, 92 patients at 3-month follow-up, and 78 patients at 12-month follow-up.

## 2. Patients and Methods

### 2.1. Patient Population

Between October 2011 and December 2012, 103 patients from 14 centers throughout Europe were enrolled in this prospective, consecutive, multicenter observational registry. All of them met the indication as listed on the products' labelling, namely,* “Patients age > 18 years presenting a mobile spinal fracture that may result from trauma (Magerl group A1, A2, or A3.1) and/or osteoporosis (IFU), with a minimum internal pedicle diameter > 5.8 mm to allow placement of the device.”* In order to ensure overall homogeneity and relevance of the results, we decided to include patients with acute fresh traumatic VCF, a traumatic fracture being defined as a fracture of the VB resulting from a high or low-energy impact occurring during a traumatic event. In this registry, data of interest (at baseline, peroperatively and 48-h postoperatively, and 3 and 12 months) were collected without requiring the physician to perform additional investigations. As surgeons must follow their own standard care practice follow-up, the patients analyzed might have no complete datasets at 3 or 12 month.

### 2.2. Ethics

Local Ethics Committee submissions were performed as per local regulations requirements. All patients gave their informed consent in accordance with ISO 14155 and the Declaration of Helsinki.

### 2.3. Operative Technique

**Figure 1:**
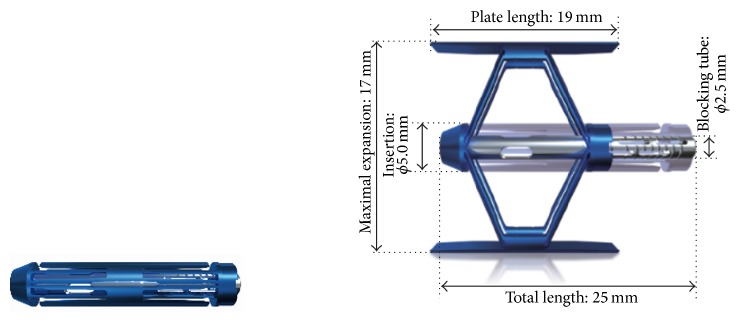
SpineJack expandable intravertebral body implant, closed and fully expanded.

The 5 mm diameter SpineJack was implanted using a percutaneous or minimally invasive posterior surgical approach using surgical tools supplied with the device [10]. With the patient being in prone position, the device was inserted into the fractured VB. Then, the implant was expanded using a specially designed tool which locks into the device and pulls the two ends of the implant towards each other. Longitudinal compression of the device causes the implant to open in the inferior-superior direction only due to the machined grooves (Figure 1).

**Figure 2:**
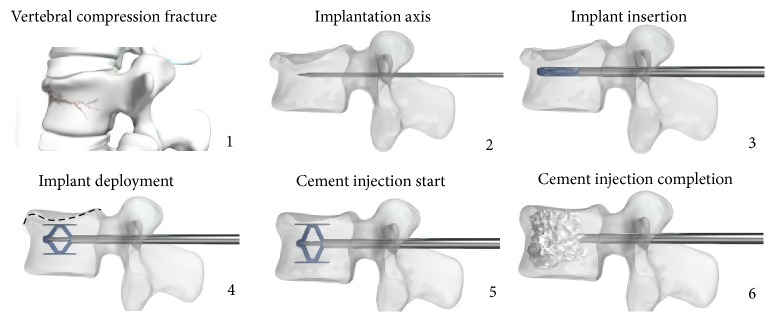
Functional principle of the SpineJack procedure.

A simple mechanism locks the implant into the desired position as controlled by the physician. Once the desired expansion obtained, the device was left in place inside the restored vertebra and polymethylmethacrylate (PMMA) bone cement was injected into and around the implant. Regular fluoroscopic controls throughout the operative procedure ensured correct implantation (Figure 2). Postoperative rehabilitation was per standard of care at the treating institution.

### 2.4. Clinical Assessment

The main outcome was pain evolution over time as assessed using a 10-cm visual analogue scale (VAS) where 0 is no pain and 10 is the worst imaginable pain. Secondary outcomes included analgesic intake, functional capacity (assessed using the self-administered Oswestry Disability Index, ODI) [11] and quality of life (assessed using the EQ-VAS from the self-administered European Quality of life-5 dimensions, EQ-5D, score) [12] evaluated at baseline, 3, and 12 months (and at discharge for analgesic intake). Complications were recorded throughout the follow-up period (adverse events device-related or not, surgery-related complications, technical incidents, device removal, subsequent compression fractures, and cement leakages).

### 2.5. Radiographic Assessment

X-rays were performed following routine clinical practice at each study site (usually 48 hours postoperatively, 3, and 12 months) and performed as per described in the protocol imaging. All available X-ray images were collected to assess the evolution of vertebral kyphotic angle.

Quantitative radiographic analysis was done on X-ray by an independent, qualified core lab using the validated FXA software developed by ACES Ing. -GmbH, Filderstadt, Germany [13]. This interim analysis focused on 48-hour, 3-month, and 12-month postoperative results.

### 2.6. Statistical Analysis

Statistical analyses were performed at the 0.05 global significance level using two-sided tests (Statistical Analysis System software, version 9.2, SAS Institute Inc., Cary, NC, USA). Within-group tests were used to test evolution between visits. Depending on the normality of the distribution, Wilcoxon's test or Student's test for pairwise comparisons were used.

Assuming a mean evolution of pain from baseline to 12 months of 5 (SD 2.5) with a precision of 0.6 and a relative precision of 12%, 66 evaluable patients were required.

Considering a lost-to-follow-up rate of 20%, 80 patients were to be included.

## 3. Results

### 3.1. Baseline Characteristics

**Figure 3:**
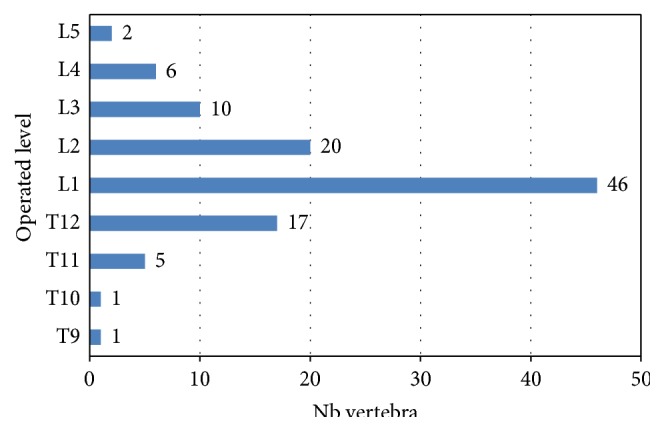
Operated vertebral levels.

The 103 patients analyzed (49.5% female) had a median age of 61.6 years, with a median BMI of 25.8 kg/m^2^. Eight patients (7.8%) presented with previous traumatic VCF; 5 of them had been already treated surgically at a level different from the one treated in this study. For 77 patients (74.8%), a previous treatment had been administered: bed rest (65.1%), bracing (9.7%), and walking aid (4.9%). A total of 108 VCF were treated (5 patients, 4.9%, had 2 fractures treated). Most fractures were due to high energy trauma (*n* = 86, i.e., 79.6% concerning 81 patients, i.e., 78.6% of population) and the remaining were traumatic fractures with associated osteoporosis (*n* = 22, i.e., 20.4% concerning 22 patients, i.e., 21.4% of population). As osteoporosis evaluation by DEXA was not performed in routine practice, it was not requested by the protocol. Osteoporotic patients were already known by the investigator, but patients with severe osteoporosis were excluded from the study. Median time from trauma to surgery was 6 days for pure traumatic fractures and 12 days in osteoporotic patients. The Magerl classification [14] showed the following distribution: 44.2% of type A3 fractures (27.9% A3.1, 4.8% A.3.2, and 11.5% A.3.3), 41.4% of type A1 fractures (1.0% A.1.3, 40.4% A1.2), 8.7% of type A2 fractures (1.0% A.2.1, 2.9% A.2.2, and 4.8% A.2.3), and 5.9% of type B fractures (2.9% B.1.3, 1.0% B.2.3, 1.0% B.3.1, and 1.0% B.3.3). Three-quarters of fractures (76.8%) were located at T12-L2 and the remaining between L3 and L5 (16.8%) or between T9 and T11 (6.4%) (Figure 3).

Mean duration of hospital stay was 4.3 ± 3.5 days. Prolonged hospitalization (27 days) was observed in one obese, osteoporotic patient treated out of IFU indications at the time of this registry as he presented with a severe A3.3 fracture. He underwent decompression and posterior instrumentation after dislocation of posterior wall at Day 4 (procedure-related); cut-out of screws in L3 occurred at 3 months, but the patient refused revision surgery. Mean follow-up period of the 103 patients was 13.1 ± 3 months.  Table 1 contains demographics and main baseline characteristics on the study population.

Twenty-three (23) patients withdrew from the study before the 12-month visit: 2 patients died due to renal failure and acute respiratory syndrome, respectively; 11 patients refused medical follow-up because of complete relief of their symptoms; 1 patient was withdrawn because of severe aggravation of a preexisting osteoporosis at inclusion, with four consecutive spontaneous fractures after surgery on Day 19, Day 49 and two fractures on Day 86; 9 patients were lost to follow-up. Flowchart of visits according to type and age of fracture is presented in Table 2.

### 3.2. Surgical Data

All surgeries were performed by surgeons, according to their standard procedures. Most fractures (94.2%) were treated under general anesthesia, 3.9% under local anesthesia, 1% by both local and general anesthesia, and the remaining 1% by spinal anesthesia. Mean duration of general anesthesia was 79.7 ± 34.7 min; all local anesthesia lasted 60 min. Of the 108 treated vertebrae, 106 (98.1%) were treated by a percutaneous approach, while 2 (1.9%) were treated by open surgery. Mean operating time was 38.3 ± 15.1 min (range: 17–105). Mean quantity of cement injected was 6.7 mL ± 2.2 mL (range: 3.0–10.8).

### 3.3. Clinical Outcome

As early as 48 hours after surgery, a significant improvement in back pain was obtained with a mean VAS score decreasing from 6.6 ± 2.6 cm at baseline to 1.4 ± 1.3 cm (mean change: −5.2 ± 2.7 cm; *p* < 0.001). This change corresponded to a median relative decrease in pain intensity of 81.5%. This improvement was maintained over the 12-month follow-up period. Mean changes in VAS scores versus baseline at 48 hours after surgery, 3 months, and 12 months are shown in Table 3.

**Figure 4:**
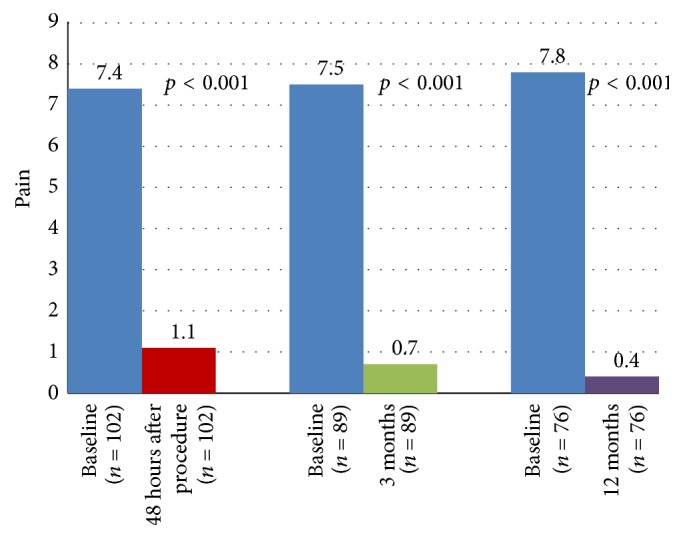
Evolution of pain: median VAS score over time.

As can be seen from Table 3, similar results were observed with both pure traumatic VCF and traumatic VCF in patients with osteoporosis. Evolution of median VAS score over time is depicted in Figure 4.

**Figure 5:**
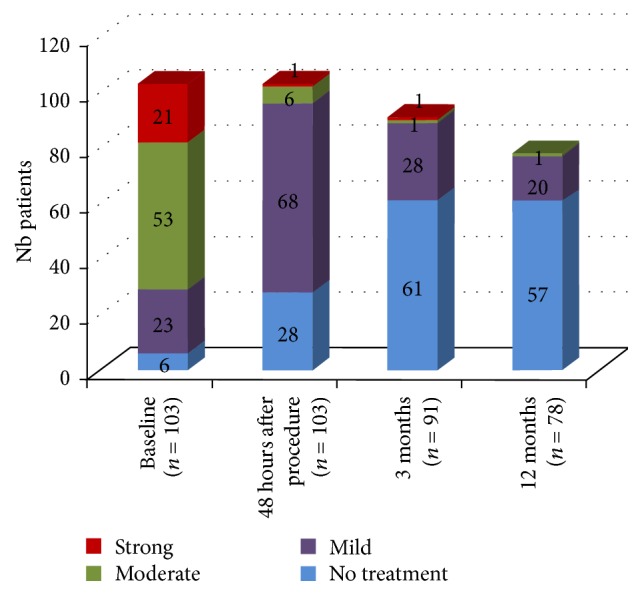
Evolution in analgesic requirements.

The decrease in pain allowed a significant reduction in the intake of analgesics within 48 hours after surgery. Indeed, before surgery, nearly three out of four patients needed strong (20.4%) or moderate (51.5%) analgesics. Two days after surgery, these percentages decreased to 1% and 5.8%, respectively. Evolution in analgesic requirement is illustrated in Figure 5.

A marked improvement in disability was observed at 3 months with a mean ODI score decreasing from 76.2 ± 20.0 at baseline to 14.2 ± 16.6 (mean change: −62.0 ± 24.9; *p* < 0.001). This change corresponded to a median relative decrease of 91.3%. This improvement was maintained at 12 months. Mean changes in ODI scores at 3 months and 12 months versus baseline are shown in Table 4.

**Figure 6:**
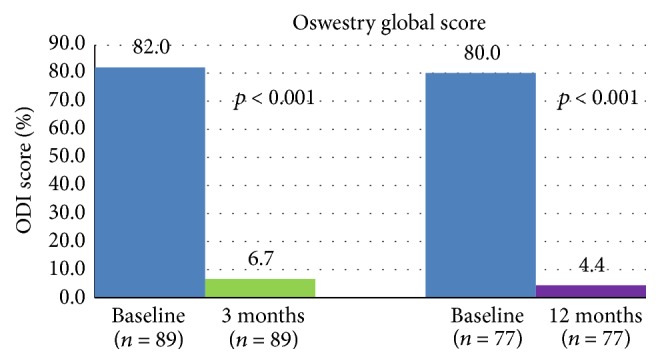
Evolution of functional disability: median ODI score over time.

As indicated in Table 4, similar results were observed with both types of fractures. Evolution of median ODI score over time is depicted in Figure 6.

A clear improvement in quality of life was observed at 3 months with a mean EQ-VAS score increasing from 53.4 ± 25.4 at baseline to 71.5 ± 24.8 (mean change: +18.1 ± 30.2; *p* < 0.001). This change corresponded to a median relative increase in quality of life of 21.1%. A slightly further improvement was observed at 12 months. Mean changes in EQ-VAS scores at 3 months and 12 months versus baseline are shown in Table 5. As indicated in this table, similar results were observed with both types of fractures.

### 3.4. Radiological Outcome

A significant and immediate reduction in the kyphotic angulation was observed 48 hours after the procedure (from 14.5 ± 8.1° to 9.2 ± 5.8°, i.e., −5.4 ± 6.3°; *p* < 0.001). Despite a lower reduction observed at 3 and 12 months, the improvement of kyphosis remained statistically significant compared to baseline (−2.5 ± 5.8° at 3 months, *p* = 0.012; −4.4 ± 6.0° at 12 months, *p* = 0.002). Similar results were observed for both types of fractures.

### 3.5. Complications

Postoperatively, 15 patients (14.6%) experienced a total of 21 adverse events which are detailed in Table 6. None of these adverse events were considered implant-related and none required device removal.

Among these adverse events, 13 serious adverse events concerning 10 patients (9.7%) were reported. Two patients died because of renal failure with lower limb vascular obliteration on Day 52 in one patient and acute respiratory syndrome at 6.8 months in the other patient.

Three patients (2.9%) experienced procedure-related complications. Eight subsequent compression fractures concerning 3 osteoporotic patients were reported. The overall adjacent fracture rate up to 1 year after surgery was 2.9% (4 fractures concerning 3 out of 103 patients). The adjacent fracture rate in the osteoporotic group was 13.6% (4 fractures concerning 3 out of 22 patients).

Cement leakage was shown in 43 out of 108 (39.8%) treated vertebrae with no clinical consequences. Leaks were detected by peroperative fluoroscopy (28.6% of cases), postoperative X-rays (28.6% of cases) or CT scans (42.8% of cases).

## 4. Discussion

Treatment of traumatic VCF without neurological symptoms and intact posterior ligament complex is still controversial. Vaccaro et al. proposed a classification system that should be helpful for decision making [15]. One major problem is that spine surgeons are faced with different classification systems for fractures [14, 16–18] and a great inhomogeneity in patient populations and treatment strategies. The Magerl classification is widely used in Europe but has shown its limits [19]. Moreover, treatment philosophies diversify impressively. In some countries, traumatic incomplete cranial burst fractures are considered to be unstable [20]. Others promote conservative treatment if posterior ligament complex and neurologic status are unaffected [15, 18]. The evidence level concerning treatment strategies is still low [21]. Yi et al. stated that there is no statistically significant difference on functional outcome two years or more after therapy between operative and nonoperative treatment [22]. Conservative treatment consisted* inter alia* of an average 4–6-week bed rest and an additional 6–12-week TLSO bracing. There is a consensus in the aims of operative treatment. Operative treatment should prevent neurologic symptoms, minimize spinal deformity and complications, allow fracture healing, and insure the best possible function.

Operative treatment ranges from combined anteroposterior approaches to minimal invasive procedures like cement augmentation [20]. Balloon kyphoplasty was used in combination with dorsal instrumentation to restore the sagittal balance [6, 23, 24]. Treatment of osteoporotic burst fractures by standalone kyphoplasty has become a standard procedure [25]. Several authors have used balloon kyphoplasty in young patients with traumatic unstable fractures [7, 26, 27]. The 10-year results of Maestretti et al. seem very promising [7]. Nevertheless, there are some studies showing that the endplate fracture reduction gained by inflation of bone tamps could not be maintained after deflation [8, 9].

The study implant is designed to work as an intravertebral reduction device directing its forces in the craniocaudal direction. The technical possibilities and improved height restoration have been shown in several biomechanical studies [28–30]. All clinical results from this study, especially the ones reported 48 h after surgery with significant reduction in pain (−81.5%) and analgesic intake (from 71.9% to 6.8% of patients requiring strong or moderate analgesics) cannot be reached by conservative treatment [4]. A marked improvement in disability (91.3% decrease in ODI score) and in quality of life (increase in 21.1% of EQ-VAS score) was obtained 3 months after surgery and maintained at 12 months. The biggest argument in favor of cement augmenting procedures in unstable fractures is the immediate pain reduction. Change in pain intensity is nearly thrice the change considered as clinically meaningful. Indeed, Ostelo et al. stated that a 30% change from baseline may be considered a clinically significant improvement [31]. Concerning radiological outcome, a significant and immediate reduction of the kyphotic angulation was observed 48 hours after surgery. Despite a lower reduction observed at 3 and 12 months, global improvement in kyphosis remained statistically significant compared to baseline.

In patients with VCF, clinical and radiological results of this minimal invasive technique have to be balanced against its complications. In the study performed by the Task Force “Wirbelsäule” (spinal column) German Society of Trauma Surgery, the complication rates (recessing and nonrecessing complications) of the different procedures varied between 14.1 and 29.7% [32–34]. Compared to these values, the procedure herein described is a safe technique. We did not observe any neurological complications. Only three patients (2.9%) experienced procedure-related complications. In patients with potential unstable fractures and poor bone quality sufficient cement has to be injected to ensure stability [29]. The adjacent fracture rate up to 1 year after surgery was 2.9% (4 fractures concerning 3 out of 103 patients). In osteoporotic patients, the risk of developing a new fracture is around 19% [35, 36]. Compared to these values, the rate of adjacent fractures seems to be low. It has to be pointed out that the average age of treated patients (59.5 years) is lower than the one reported in most studies about osteoporotic fractures. Cement leakage was detected in 39.8% of treated vertebrae with no clinical consequences. These numbers are comparable to other studies on unstable fractures treatment [25]. Symptomatic cement leakages were not seen in any patient.

## 5. Conclusion

The present study supports the growing interest in minimally invasive techniques in the management of spinal injuries with no neurological deficit. These long-term results confirm the stability of the correction over time. The device described allows an effective, low-risk procedure for patients with vertebral fractures of traumatic origin with a significant reduction in pain and analgesic consumption achieved immediately after surgery and maintained over time. Additionally, this procedure allows a fast and sustainable improvement in quality of life. Main complications included asymptomatic cement extravasation and adjacent fractures, which were caused by the cementation technique or resulted from underlying osteoporosis but were not due to the procedure itself. One-year results from this registry are very promising and will be confirmed with the 2-year outcomes and have to be further proven by comparative randomized study results.

## Figures and Tables

**Table 1:** Baseline patient characteristics.

	Traumatic VCF with osteoporosis	Traumatic VCF	Total
	*n* = 22	*n* = 81	*n* = 103
Age, mean (SD) (years)	69.2 (12.1)	59.5 (15.5)	61.6 (15.3)
Female, *n* (%)	20 (90.9%)	31 (38.3%)	51 (49.5%)
BMI, mean (SD) (kg/m^2^)	25.9 (4.6)	25.8 (3.8)	25.8 (3.9)
Number of augmented vertebrae	22	86	108
Time from fracture to procedure, mean (SD) (days)	13.9 (8.9)	8.1 (7.1)	9.2 (7.8)
Pain, VAS score, mean (SD) (cm)	6.8 (1.9)	6.6 (2.8)	6.6 (2.6)
ODI score, mean (SD)	70.5 (16.0)	78.3 (19.9)	76.6 (19.4)
EQ-VAS	47.7 (21.4)	51.1 (26.6)	50.4 (25.5)

**Table 2:** Flowchart of visits according to type and age of fracture.

Inclusion					
*n* = 103					
Traumatic and osteoporosis associated			Traumatic only		
*n* = 22			*n* = 81		
<15 days	[15; 30] days	[30; 45] days	<15 days	[15; 30] days	[30; 45] days
*n* = 14	*n* = 7	*n* = 1	*n* = 70	*n* = 9	*n* = 2

Procedure					
*n* = 103					
Traumatic and osteoporosis associated			Traumatic only		
*n* = 22			*n* = 81		

<15 days	[15; 30] days	[30; 45] days	<15 days	[15; 30] days	[30; 45] days
*n* = 14	*n* = 7	*n* = 1	*n* = 70	*n* = 9	*n* = 2

48 hours after surgery					
*n* = 103					
Traumatic and osteoporosis associated			Traumatic only		
*n* = 22			*n* = 81		

<15 days	[15; 30] days	[30; 45] days	<15 days	[15; 30] days	[30; 45] days
*n* = 14	*n* = 7	*n* = 1	*n* = 70	*n* = 9	*n* = 2

3 months after surgery					
*n* = 92					
Traumatic and Osteoporosis associated			Traumatic Only		
*n* = 21			*n* = 71		

<15 days	[15; 30] days	[30; 45] days	<15 days	[15; 30] days	[30; 45] days
*n* = 13	*n* = 7	*n* = 1	*n* = 63	*n* = 6	*n* = 2

12 months after surgery					
*n* = 78					
Traumatic and Osteoporosis associated			Traumatic Only		
*n* = 16			*n* = 62		

<15 days	[15; 30] days	[30; 45] days	<15 days	[15; 30] days	[30; 45] days
*n* = 11	*n* = 4	*n* = 1	*n* = 53	*n* = 7	*n* = 2

**Table 3:** Absolute changes (cm) and relative (%) changes in VAS score.

	Traumatic VCF with osteoporosis	Traumatic VCF	Total
48 h versus baseline	*n* = 22	*n* = 80	*n* = 102

Mean (SD) absolute changes	−5.5 (1.9)	−5.1 (2.9)	−5.2 (2.7)
Median absolute changes	−5.7	−6.1	−6.0
Within-group test	<.001 (Student)	<.001 (Wilcoxon)	<.001 (Wilcoxon)
Median relative changes	−81.0	−81.8	−81.5

3 months versus baseline	*n* = 20	*n* = 69	*n* = 89

Mean (SD) absolute changes	−5.5 (2.7)	−5.3 (3.0)	−5.3 (2.9)
Median absolute changes	−6.2	−6.0	−6.0
Within-group test	<.001 (Student)	<.001 (Wilcoxon)	<.001 (Wilcoxon)
Median relative changes	−90.0	−88.0	−88.0

12 months versus baseline	*n* = 16	*n* = 60	*n* = 76

Mean (SD) absolute changes	−5.7 (2.3)	−5.5 (3.0)	−5.5 (2.9)
Median absolute changes	−5.2	−6.3	−5.8
Within-group test	<.001 (Student)	<.001 (Wilcoxon)	<.001 (Wilcoxon)
Median relative changes	−90.3	−92.2	−91.5

**Table 4:** Absolute and relative (%) changes in ODI score.

	Traumatic VCF with osteoporosis	Traumatic VCF	Total
3 months versus baseline	*n* = 20	*n* = 69	*n* = 89

Mean (SD) absolute changes	−54.3 (26.6)	−64.3 (24.1)	−62.0 (24.9)
Median absolute changes	−62.0	−73.3	−71.3
Within-group test	<.001 (Student)	<.001 (Wilcoxon)	<.001 (Wilcoxon)
Median relative changes	−91.0	−92.7	−91.3

12 months versus baseline	*n* = 16	*n* = 61	*n* = 77

Mean (SD) absolute changes	−60.7 (18.8)	−67.1 (24.9)	−65.7 (23.8)
Median absolute changes	−64.1	−74.0	−73.3
Within-group test	<.001 (Student)	<.001 (Wilcoxon)	<.001 (Wilcoxon)
Median relative changes	−92.9	−95.0	−94.9

**Table 5:** Absolute and relative (%) changes in EQ-VAS score.

	Traumatic VCF with osteoporosis	Traumatic VCF	Total
3 months versus baseline	*n* = 20	*n* = 66	*n* = 86

Mean (SD) absolute changes	23.1 (25.3)	16.6 (31.6)	18.1 (30.2)
Median absolute changes	16.5	12.5	13.0
Within-group test	<.001 (Student)	<.001 (Student)	<.001 (Wilcoxon)
Median relative changes	27.0	17.4	21.1

12 months versus baseline	*n* = 16	*n* = 59	*n* = 75

Mean (SD) absolute changes	29.3 (22.3)	23.4 (28.4)	24.6 (27.2)
Median absolute changes	26.5	16.0	19.0
Within-group test	<.001 (Student)	<.001 (Wilcoxon)	<.001 (Wilcoxon)
Median relative changes	44.2	33.3	38.3

**Table 6:** Adverse events.

Patient number	Type of event	Time after surgery (days)	Relationship to device	Relationship to procedure	Outcome at 12 M
11R01	Death due to acute kidney failure aggravation with vascular obliteration of leg	52	No	No	Death

11R04	Sigma diverticulitis (preexisting condition at inclusion) aggravation: hospitalization for diverticulitis resection	8	No	No	Resolved

11R07	Asymptomatic adjacent vertebral fracture T11: No treatment needed (treated vertebra T12)	99	No	Yes	Ongoing (fracture remains stable)

13R06	Prolongation of hospitalization for dislocation of posterior wall secondary to surgery which leads to sensorial deficit → surgery with wrong indication	4	No	Yes	Resolved
	Breakage of screws in L3: recommendation of revision surgery but patient declined surgery	82	No	No	Ongoing Patient lost to follow-up

31R04	Posterior articular conflict due to a discopathy L4/L5 grade IV associated to a degenerative spondylolisthesis grade II Meyerding	147	No	No	Resolved at Day 455

31R05	Algoneurodystrophic syndrome due to calcaneum fracture resolved by decompressive surgery	100	No	No	Resolved

31R06	Prostatic cancer	257	No	No	Ongoing

31R15	Bleeding at the point of the skin incision just after the surgery	2	No	No	Resolved

31R16	Spontaneous adjacent fracture T12 (treated vertebra L1) in a context of major osteoporosis aggravation: hospitalization for vertebroplasty	19	No	No	Resolved
	Spontaneous adjacent fracture L2 (treated vertebra L1) in a context of major osteoporosis aggravation treatment: analgesics and Zoledronic acid monohydrate	49	No	No	Patient discontinued
	Spontaneous new fracture L4 (treated vertebra L1) in a context of major osteoporosis aggravation treatment: analgesics and Zoledronic acid monohydrate	86	No	No	Patient discontinued
	Spontaneous new fracture L5 (treated vertebra L1) in a context of major osteoporosis aggravation treatment: analgesics and Zoledronic acid monohydrate	86	No	No	Patient discontinued

35R08	Death due to acute respiratory syndrome	204	No	No	Death

35R11	Hospitalization in psychiatric department	Not available	No	No	Resolved

36R01	Subsequent fracture at T12 level (previous treated level L2): hospitalization for vertebroplasty	18	No	No	Resolved
	Subsequent fracture at T11 level (previous treated level L2): hospitalization for vertebroplasty	126	No	No	Resolved
	Adjacent fracture at L3 level (previous treated level L2): hospitalization for vertebroplasty	126	No	No	Resolved

36R02	Lumbar pain	418	No	No	Resolved

38R07	Shoulder fracture due to a fall discovered lately and treated by physiotherapy	276	No	No	Ongoing

41R02	Collapse of treated vertebral body associated with canal compromise and hematoma leading to neurological symptoms	16	No	Yes	Improved
